# Mind the Gap: A Questionnaire on the Distance between Diagnostic Advances and Clinical Practice in Skin Cancer Treatment

**DOI:** 10.3390/medicina60010155

**Published:** 2024-01-15

**Authors:** Giuseppe Diluiso, Mirco Pozzi, Flavio Giulio Liso, Vanessa Marron Mendes, Jenna Hannouille, Luigi Losco, Alberto Bolletta, Emanuele Cigna, Michela Schettino

**Affiliations:** 1 Unit of Plastic Surgery, Department of Medicine, Surgery and Neuroscience, University of Siena, 53100 Siena, Italy; giuseppe.diluiso@student.unisi.it (G.D.); mirco.pozzi@student.unisi.it (M.P.); 2 Independent Researcher, 76123 Andria, Italy; flaviogl@hotmail.it; 3 Service de Chirurgie Plastique, Hôpital CHIREC (Braine L’Alleud-Waterloo, Belgium), 1420 Braine-L’Alleud, Belgium; vanessa.marronmendes@chirec.be (V.M.M.); michelaschettino@gmail.com (M.S.); 4 Hôpital Delta (Bruxelles), ULB—Université Libre de Bruxelles, 1050 Bruxelles, Belgium; jenna.hannouille@ulb.be; 5 Plastic Surgery Unit, Department of Medicine, Surgery and Dentistry, University of Salerno, Baronissi, 84081 Salerno, Italy; 6 Plastic Surgery and Microsurgery Unit, Department of Translational Research and New Technologies in Medicine and Surgery, University of Pisa, 56126 Pisa, Italy; alberto.bolletta@unipi.it (A.B.); emanuele.cigna@unipi.it (E.C.)

**Keywords:** surgery, plastic, search engine, dermatology, surgeons, Europe, software, surveys and questionnaires, dermato-oncology

## Abstract

*Background and Objectives*: Significant progress has been made in skin cancer diagnosis, with a surge in available technologies in recent years. Despite this, the practical application and integration of these technologies in dermatology and plastic surgery remain uneven. *Materials and Methods*: A comprehensive 20-question survey was designed and distributed using online survey administration software (Google Forms, 2018, Google, Mountain View, CA, USA) from June 2023 to September 2023. The survey aimed to assess the knowledge and utilization of dermatologic diagnostic advancements among plastic surgeons in various European countries. *Results*: Data were obtained from 29 plastic surgeons across nine European countries, revealing a notable gap between diagnostic technologies and their routine use in surgical practice. The gap for some technologies was both cognitive and applicative; for electrical impedance spectroscopy (EIS) and multispectral imaging, only 6.9% of the sample knew of the technologies and no surgeons in the sample used them. In the case of other technologies, such as high-frequency ultrasound (HFUS), 72.4% of the sample knew about them but only 34.5% used them, highlighting a more significant application problem. *Conclusions*: Spotlighting this discrepancy provides a valuable foundation for initiating collaborative efforts between units and facilitating knowledge exchange among diverse specialists. This, in turn, contributes to advancing clinical practice by integrating the innovative opportunities presented by ongoing research.

## 1. Introduction

The incidence of skin cancer diagnoses globally is steadily rising due to an aging population and advances in diagnostic techniques [1].

Approximately one in three cancers diagnosed worldwide are skin cancers, with an estimated 2–3 million Nonmelanoma Skin Cancers (NMSCs) and 132,000 Melanoma Skin Cancers (MSCs) diagnosed annually [2]. Despite these alarming numbers, the integration of novel diagnostic approaches into clinical practice has been slow. Other data concerning the United States and Europe are even more impressive: 247,894 NMSC cases and 101,507 MSC cases in the European Union for the year 2022 [3] and 97,610 MSC cases in the United States for the year 2023 [4]. Moreover, especially with regard to NMSCs, the number appears to be greatly underestimated; actually, in some health systems, there is no requirement to report this type of diagnosis.

In recent years, technological advancements have transformed skin cancer diagnosis, moving from subjective clinical assessments to computer-assisted or artificial intelligence-driven systems. However, these advancements have not seamlessly integrated into everyday clinical practice, especially in smaller health care settings [5]. These systems compile a vast array of global data by accumulating the experience of numerous centers, resulting in sensitivity levels approaching 100 percent and specificities exceeding 90 percent.

The future looks promising as we steadily move closer to a more objective form of diagnosis. However, these diagnostic advancements are not accompanied by parallel progress in everyday clinical practice. It is important to note that these technologies primarily impact larger hospital and university settings and are less readily adaptable in smaller contexts [6].

This article addresses the noticeable gap between diagnostic advancements and daily surgical practices in the field of plastic surgery, where plastic surgeons often lack updated knowledge and access to cutting-edge diagnostic tools. To objectify this reflection, we focused our analysis on the knowledge and application of several diagnostic applications [7,8] (Table 1):

− Adhesive patch biopsy (tape stripping mRNA);
− EIS (electrical impedance spectroscopy);
− Multispectral imaging;
− High-frequency ultrasonography (HFUS);
− Optical coherence tomography (OCT);
− Reflectance confocal microscopy (RCM);
− Artificial intelligence (AI) and computer analysis.

These various diagnostic instruments are useful in the diagnosis or indication for biopsy of suspicious skin neoformations for MSCs and NMSCs and have been newly introduced in dermato-oncology practice. Next, we analyzed their prevalence in the field of dermato-oncology in plastic surgery.

## 2. Materials and Methods

A 20-question survey was designed collaboratively by plastic surgeons and dermatologists actively engaged in dermato-oncologic surgery. The survey aimed to assess the understanding and utilization of diagnostic innovations in oncodermatologic surgery among plastic surgeons.

The purpose of the questionnaire was to assess, among an initial sample of plastic surgeons distributed throughout Europe, the level of understanding and effective utilization of diagnostic innovations in oncodermatologic surgery. The questionnaire was distributed by personal invitation through local and international networks to several members of national and international plastic surgery scientific societies to ascertain the perception, knowledge, and application of new diagnostic tools by plastic surgeons.

The surgeons contacted were all part of institutions considered to be referral centers for the treatment of cutaneous neoplasms. The inclusion criteria for these centers were the presence of a plastic surgery department and a dermatology department in the same hospital and the treatment of all cutaneous neoplasms (both NMSCs and MSCs), apart from sarcomas, the treatment of which is usually the responsibility of dedicated orthopedic teams (Table 2).

The survey was distributed anonymously to 50 plastic surgeons in nine European countries, all affiliated with institutions considered referral centers for cutaneous neoplasms. Twenty-nine complete questionnaires were obtained and rigorously analyzed. The questionnaire consisted of a total of 20 questions, with the first section focusing on physicians’ knowledge of diagnostic advances in oncodermatology and their involvement in treating patients. The second section addressed the routine use of emerging technologies in their daily practice, while the third section delved into the degree of collaboration with dermatology departments for skin cancer diagnosis and preoperative evaluations.

In addition, participants were asked to provide their perspectives on potential future applications of these new technologies, should they be available in their centers.

We selected a sample of surgeons to assay the knowledge and use of these technologies by plastic surgery specialists. We did not select dermatologists because, in this case, the problem would be mainly one of updating; as for surgeons, we essentially analyzed the distance between these professionals and the diagnostic advances in the field of oncologic dermatology. In fact, while the concept of division of expertise remains intact, the work of surgeons is strongly influenced by the lack of knowledge or application of these techniques.

Subsequently, the responses were meticulously reviewed and validated by the expert panel. This study was conducted in accordance with the Declaration of Helsinki. Personal data were processed in accordance with the European General Data Protection Regulation. Data were analyzed anonymously. Participants were informed in detail about the scope of the study and provided informed consent before initiation.

## 3. Results

### 3.1. General Results

Out of the 29 plastic surgeons surveyed, 48.3% performed more than ten oncodermatology surgeries per week, highlighting the active involvement of plastic surgeons in treating skin cancers. However, only 48.3% of these centers offered dermoscopy services in daily clinical practice.

### 3.2. Knowledge of Diagnostic Instruments

**Figure 1:**
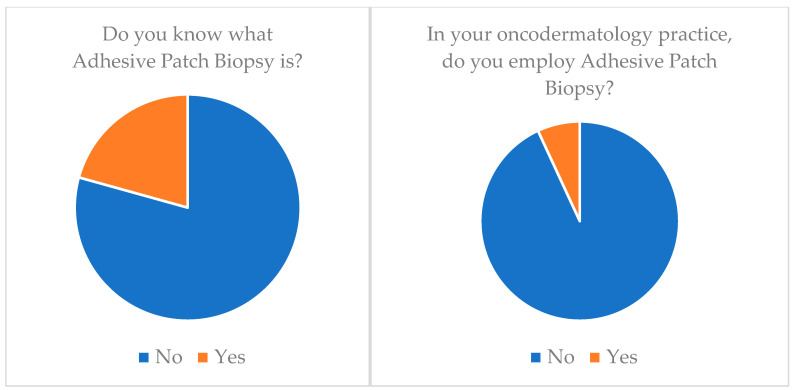
Adhesive patch biopsy (tape stripping mRNA) knowledge and application.

**Figure 2:**
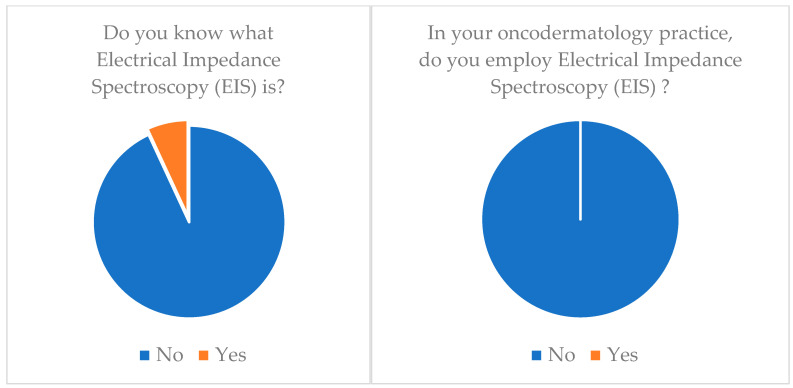
EIS (electrical impedance spectroscopy) knowledge and application.

**Figure 3:**
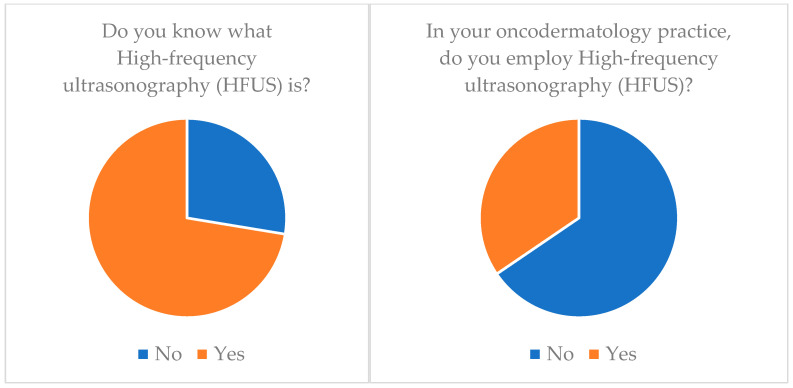
High-frequency ultrasonography (HFUS) knowledge and application.

**Figure 4:**
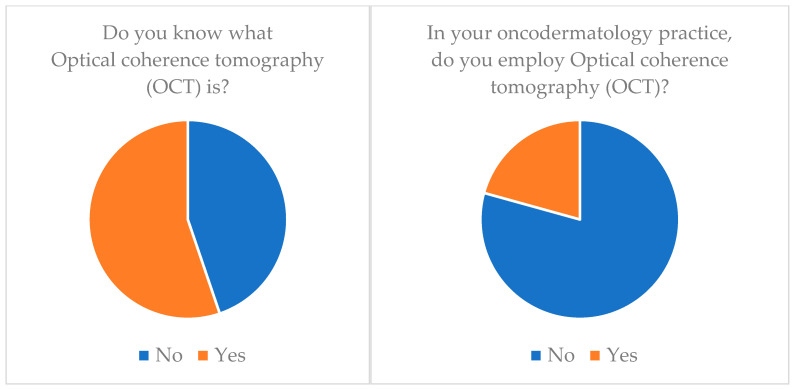
Optical coherence tomography (OCT) knowledge and application.

- **Adhesive patch biopsy (tape stripping mRNA)**: Only 20.7% (*n* = 6) of our cohort knew about this technology and only 6.9% (*n* = 2) applied it (Table 3) (Figure 1).
- **EIS (electrical impedance spectroscopy)**: Just 6.9% (*n* = 2) of our cohort knew about this technology and none of them applied it (*n* = 0) (Table 3) (Figure 2).
- **Multispectral imaging**: Just 6.9% (*n* = 2) of our cohort knew about this technology and none of them applied it (*n* = 0) (Table 3).
- **High-frequency ultrasonography (HFUS)**: 72.4% (*n* = 21) of our cohort knew about this technology but only 34.5% (*n* = 10) applied it (Table 3) (Figure 3).
- **Optical coherence tomography (OCT)**: 55.2% (*n* = 16) of our cohort knew about this technology but only 20.7% (*n* = 6) applied it (Table 3) (Figure 4).
- **Confocal reflectance microscopy (RCM)**: Only 55.2% (*n* = 16) of our cohort knew about this technology and only 20.7% (*n* = 6) applied it (Table 3).
- **Artificial intelligence (AI) and computer analysis**: 72.4% (*n* = 21) of our cohort knew about this technology and only 13.8% (*n* = 4) applied it (Table 3).

In the case of skin cancer diagnosis, 72.4% (*n* = 21) of plastic surgery departments engaged in collaboration with a dermatology unit. However, when it comes to the preoperative evaluation of excision margins, only 27.6% (*n* = 8) of them worked in conjunction with dermatologists.

Regarding the consideration of these new diagnostic tools, they were considered useful in the totality of our sample. Of these, 34.5% (*n* = 10) said they planned to apply them in the future, 58.6% (*n* = 17) said they would employ them if they had them available at their center, and only 6.9% (*n* = 2) said they employed them regularly.

## 4. Discussion

The data from our sample quite clearly express the gap between surgical practice and diagnostic techniques (both basic techniques such as dermoscopy and more advanced ones). Although we have units that consistently practice dermatologic surgery, only 51.7% provide a dermoscopy service during daily clinical practice.

The study underscores a disconnect between surgical practices and diagnostic advancements, with only half of the surveyed plastic surgeons offering dermoscopy services. Despite awareness of technologies like high-frequency ultrasonography, optical coherence tomography, and artificial intelligence, their routine use in daily practice was limited.

Conversely, these methods would be particularly useful in different clinical scenarios. Here we explain its characteristics more extensively (Table 4).

**Adhesive patch biopsy** (tape stripping mRNA) represents a noninvasive diagnostic technique with enormous potential and ease of use [9,10,17,18,19,20,21,22,23,24,25,26] (Table 5). Studies have focused on its utility in early melanoma diagnosis and prognostic definition, replacing or even in combination with classical histology. It is a procedure that involves simple taping of the pigmented neoformation and detection of the mRNA present in the stratum corneum; although there is no nucleus in this cell layer, in the case of neoplasia there is expression of these molecules through mechanisms not yet fully elucidated.

In this way, the presence of mRNA bound to different genes is detected; several proposals have been made on the batteries of genes to be analyzed. The use of 17 genes proposed by Wachsman provided 100% sensitivity and 88% specificity [19].

Gerami et al. proposed using only two genes (LINC00518 and PRAME) with a sensitivity of 91% and specificity of 69%, a method known as PLA (pigmented lesion assay) [17,18].

In contrast, the review by Thomsen et al. [9] indicates a sensitivity of 86.9% and specificity of 82.4% for a two-gene assay (LINC00518 and PRAME), while in an analysis of studies that included other genes, the sensitivity and specificity values ranged from 68.8 to 100 percent and 69.1 to 100 percent, respectively.

From an economic point of view, moreover, the method has costs comparable to those of classical histological examinations according to some authors, while according to a study by Hornberger, the economic savings of using PLA compared to a visualization and surgical biopsy procedure with histological examination would be 47% [24].

It thus represents an additional resource that can avoid unnecessary excisional biopsies and improve clinical and prognostic framing by detecting the presence of several genes.

**EIS (electrical impedance spectroscopy)** [5,21,27,28,29,30] (Table 5) is a method that studies changes in tissue electrical impedance, a value that changes as the histological characteristics of the neoplasm change. The variation in this value depends on tissue cellularity and water content. It should not be understood as a primarily diagnostic tool, but as an auxiliary tool in the choice between excision and observation; it can detect both MSCs and NMSCs, although it has critical issues with regard to the differential diagnosis of seborrheic keratoses and other limitations related to the presence of ulcerated or bleeding lesions and the variation in values related to skin characteristics in different body areas. It also has high sensitivity in the diagnosis of melanoma but significantly lower specificity.

**Multispectral imaging** (Table 5), on the other hand, is a noninvasive and fully automated lesion analysis tool.

The most studied device using this technology is the MelaFind (STRATA Skin Sciences; MELA Sciences Inc., Irvington, NY, USA) [11]. This instrument allows imaging up to 2.5 mm below the skin surface and analyzes 10 digital images by scanning the light reflectance distribution pattern.

It appears to possess high sensitivity (98.3%) but low specificity (9.9%), although the data vary widely among studies; its role should be to assist clinical and dermoscopy decision making, not to replace it. The ratio of MSC to benign pigmented lesions is 1:8–1:10, which coincides with that of good dermoscopy combined with clinical assessments.

The picture is slightly different regarding **high-frequency ultrasound (HFUS)** [31,32,33] (Table 5). In fact, only 27.6 percent of the sample did not know what it was, but in this case, despite knowledge, 65.5 percent of the sample did not employ it in daily practice (Table 2).

HFUS is an imaging technique that uses high-frequency sound waves to produce high-resolution, real-time images of tissues below the skin surface to a depth of about 15 mm. It is particularly useful for assessing the depth and characteristics of skin lesions. It provides valuable information about the extent of tumor infiltration and helps plan surgical procedures.

HFUS helps dermatologists and plastic surgeons determine the appropriate treatment approach for skin cancers and other dermatologic conditions.

Indeed, among the advantages of this procedure is that it provides different patterns depending on the lesions, thus being applicable as a diagnostic aid for both MSCs and NSCs and other types of skin pathology. Its disadvantages, on the other hand, include the fact that it is an operator-dependent method and lacks the ability to distinguish the cellularity of the tissue; for these reasons, although it is of great interest, it is not yet a tool capable of making diagnoses and is not able to replace histology.

**Optical coherence tomography (OCT)** [34,35,36] (Table 5) and confocal reflection microscopy (RCM), on the other hand, were known to only part of the sample, but their use was still minimal (employed by only 20.7% of our cohort). Optical coherence tomography (OCT) is a noninvasive imaging technique that uses light waves to capture detailed, high-resolution cross-sectional images of biological tissues, including skin. It operates on the principle of measuring the time delay of reflected light to create detailed images of tissue microstructures. It allows clinicians to visualize skin layers and structures in real time. It is particularly useful for assessing skin lesions, measuring their depth, and differentiating between benign and malignant skin conditions. OCT provides valuable information for diagnosis and treatment planning.

On the other hand, **reflectance confocal microscopy (RCM)** [37,38] (Table 5) is an advanced imaging technique that enables real-time, high-resolution visualization of skin at the cellular level. It utilizes a laser to capture images of skin tissue by measuring the reflection of light from different depths within the skin. RCM is particularly valuable in dermatology for in vivo diagnosis of skin lesions. It allows dermatologists to examine cellular structures, identify abnormal cells, and assess the margins of skin lesions. RCM aids in the early detection and accurate diagnosis of skin cancers, including melanoma. The possibilities of this technology are remarkable; in fact, it allows in vivo histology, enabling the patterns of different skin lesions to be distinguished; the disadvantages, however, are many, starting with the depth of analysis, which is limited to the papillary dermis (about 200 μm), followed by the cost of the machine and the limited dissemination and expertise in reading the results.

Finally, most of the sample said they knew what machine learning and computer diagnostics are, but at the same time many of them did not employ them in their daily practice. **Artificial intelligence (AI)** [39,40] (Table 5) and computer-based diagnosis refer to the integration of machine learning algorithms and computer technology in oncodermatology. These systems analyze and interpret dermatological images, including photographs and histopathological slides, to assist in diagnosing skin conditions. AI and computer-based diagnosis can rapidly process large datasets, identify patterns, and provide diagnostic recommendations. These tools are increasingly used in dermatology for the early detection of skin cancers, such as melanoma, and for the automated analysis of skin lesions. They enhance diagnostic accuracy and efficiency in clinical practice.

Further thought needs to be given to the possibility of applying machine learning and artificial intelligence to smartphone applications. The use of smartphone applications to diagnose skin lesions by capturing them with increasingly better-resolution cameras is not a new idea. A number of apps capable of making diagnoses have long been placed in app stores, but often these apps have turned out to be unsupported by real scientific evidence, while still others were based on partly outdated basic principles, such as the ABCDE criteria [39].

The basic idea, however, is intriguing, and the use of artificial intelligence and machine learning could enable the development of more reliable applications supported by stronger evidence.

At the same time, it would allow remote dermatology consultations to be provided with greater confidence, expanding treatment options in economically disadvantaged or rural settings [6,41].

**Table 5:** Some of the main and more recent studies on reviewed techniques.

Author	Year	Journal	Methodology	Techniques
Lassau et al. [33]	1997	*Radiographics*	Original Article	HFUS
Moncrieff et al. [22]	2002	*Br J Dermatol*	Original Article	MI
Bessoud et al. [12]	2003	*Ultrasound Med Biol*	Original Article	HFUS
Glickman et al. [42]	2003	*Skin Res Technol*	Original Article	EIS
Ruocco et al. [43]	2004	*Dermatol Surg*	Review Article	RCM
Har-shai et al. [44]	2005	*Plast Reconstr Surg*	Multicenter Study	EIS
Machet et al. [45]	2009	*Ultrasound Med Biol*	Review Article	HFUS
Wachsman et al. [19]	2011	*Br J Dermatol*	Original Article	APB
Monheit et al. [11]	2011	*Arch Dermatol*	Original Article	MI
Crisan et al. [31]	2013	*Arch Dermatol Res*	Original Article	HFUS
Gerami et al. [10]	2014	*J Am Acad Dermatol*	Original Article	APB
Malvehy et al. [5]	2014	*Br J Dermatol*	Clinical Trial	MI
Meyer et al. [46]	2014	*Br J Dermatol*	Original Article	HFUS, OCT
Longo et al. [47]	2014	*J Am Acad Dermatol*	Original Article	RCM
March et al. [8]	2015	*J Am Acad Dermatol*	Review Article	EIS, MI
Markowitz et al. [13]	2015	*J Clin Aesthet Dermatol*	Original Article	OCT
Gambichler et al. [14]	2015	*J Eur Acad Dermatol Venereol*	Original Article	OCT
Olsen et al. [34]	2016	*Photodiagn Photodyn Ther*	Original Article	OCT
Que et al. [15]	2016	*Dermatol Clin*	Original Article	RCM
Borsari et al. [16]	2016	*JAMA Dermatol*	Original Article	RCM
Guilera et al. [48]	2016	*Dermatol Clin*	Review Article	RCM
Ferris et al. [17]	2017	*JAMA Dermatol*	Original Article	APB
Gerami et al. [18]	2017	*J Am Acad Dermatol*	Original Article	APB
Welzel et al. [28]	2017	*J Dtsch Dermatol Ges*	Review Article	EIS, OCT, RCM, MI
Braun et al. [29]	2017	*Dermatol Clin*	Original Article	EIS
Niculescu et al. [49]	2017	*Photodiagn Photodyn Ther*	Original Article	OCT
Heibel et al. [7]	2020	*Am J Clin Dermatol*	Review Article	APB; EIS, MI; HFUS; OCT; RCM
Chu et al. [39]	2020	*Front Med*	Review Article	AI
Pathania et al. [25]	2022	*J Cosmet Dermatol*	Review Article	APB; EIS, MI; HFUS; OCT; RCM: AI
Owida et al. [50]	2022	*J Skin Cancer*	Review Article	MI; HUFS; OCT; RCM
Thomsen et al. [9]	2023	*Skin Res Technol*	Systematic Review	APB

APB: adhesive patch biopsy; EIS: electrical impedance spectroscopy; MI: multispectral imaging; HFUS: high-frequency ultrasonography; OCT: optical coherence tomography; RCM: reflectance confocal microscopy; AI: computer-assisted diagnosis and artificial intelligence.

Our sample data reveal a significant disconnection between dermatological and surgical practices. To illustrate this point, dermoscopy is available in only 50% of cases, with collaboration with dermatology units for diagnosis occurring in 72.4% of cases but only in 27.6% of cases for preoperative margin assessment. Clearly, there exists a noticeable gap in health care practices [31,51].

Although theoretically available, these technologies are not being applied due to lack of communication and knowledge integration. Today, health care systems tend to separate the clinical and diagnostic aspects from the surgical procedure required for the same pathology.

From the perspective of plastic surgeons, moreover, the approach to pathology is often surgical even when it may not be necessary. Indeed, the possibilities and reconstructive capabilities of dermato-oncology drive the surgeon to an often more aggressive approach than the dermatologist. Greater integration here, too, would lead to better selection of surgical patients; for example, consider the 1:8 ratio in diagnoses of excised melanomas versus excised benign pigmented lesions of experienced dermatologists compared with the ratio of more than 1:30 of other specialists such as plastic surgeons [7]. In plastic surgery, moreover, functionally and aesthetically adequate reconstruction is the standard of care [52,53,54,55]. Increasing attention is being paid to decreasing the surgical impact, both demolitive and reconstructive, for the patient, seeking solutions that are satisfactory in terms of form and function and minimally invasive and debilitating. An example of this trend comes to us from orthoplastic and breast oncoplasty, which represent two important examples of the development of plastic surgery from the perspective of knowledge integration [56,57].

### 4.1. Skin Cancer Units

Disconnection of expertise is not uncommon in medicine; the distance and compartmentalization of expertise has been addressed before, as seen in the case of breast cancer. The creation of Breast Units, for example, stems from the need to centralize expertise within larger, more experienced centers and to coordinate the various clinical specialists needed for comprehensive management of the disease. Moreover, the criteria for establishing these centers are particularly rigorous and complex, thus ensuring better quality of care. Similarly, centralization is also desirable for an extremely prevalent disease such as skin cancer. Some examples of Skin Cancer Units already exist, but they are not yet sufficiently widespread. Similar reasoning has also been applied by some to the teaching of the subject, which should be considered as a single disease and not as two separate entities [58].

In our idea, this model of development should provide for common programs in the training pathway that would allow plastic surgeons, dermatologists, oncologists, radiotherapists, immunotherapists, and all the figures who revolve around the diagnosis and treatment of cutaneous oncologic pathology to work together during training, developing knowledge and awareness of the possibilities offered by the work of their colleagues.

This would lead to growth in terms of integration and interchange of knowledge; indeed, the different figures would be accustomed to calling on the professionalism of their colleagues knowing well the type of work or procedure needed on a case-by-case basis.

The centrality of training would thus shift from a “ specialist” to a “ pathological” view, while maintaining the profile of expertise and ultra-specialization needed to achieve optimal results.

The presence of dedicated units specializing in the treatment of cutaneous oncologic pathology, rather than the presence of plastic surgery or dermatology units dealing with it, underlies the same idea: responding quickly, effectively, and all-inclusively to the practical and clinical needs of the patient, rather than remaining anchored in our idea of specialization. We reiterate how this reasoning of ours does not challenge the system of specialization but proposes its better practical application.

This model of development also sees many difficulties in its application in relation to the material and economic possibilities of different health care systems and responds to health policy logic that is beyond the scope of our discussion.

**Figure 5:**
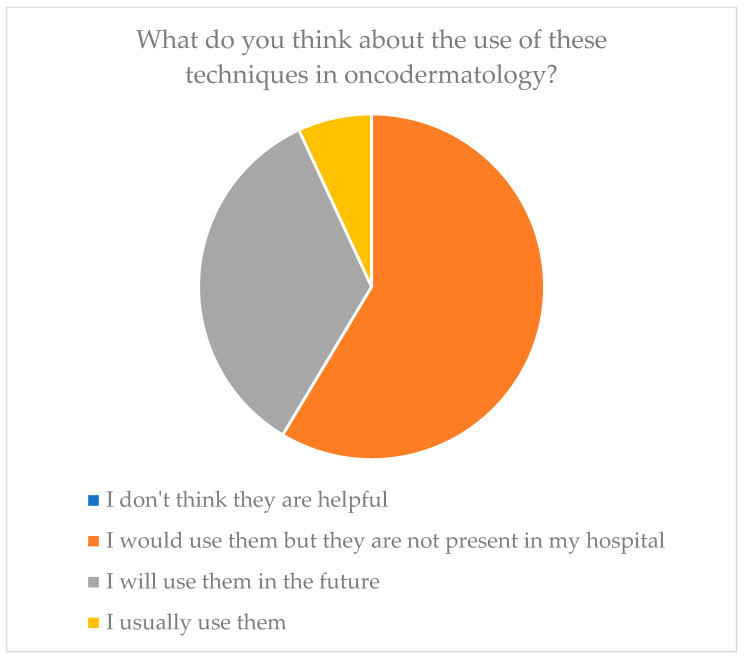
Future resolutions of plastic surgeons toward these technologies.

As evidence of this reasoning and the need to integrate knowledge, it is sufficient to analyze the responses given in our sample regarding what people think about the use of these technologies in dermato-oncology surgery. The entire sample believed that these technologies are useful, and 58.6% of the sample said they would employ them if only they had the opportunity (Figure 5).

It is also interesting to consider the duplicity of this gap. On the one hand, there is a cognizance gap; essentially, the surgeon does not know of the available technologies. Second, there is an application gap that lies in the material lack of these devices. Interesting examples of this dual gap are OCT and HFUS; in fact, 55.2% of the sample knew what OCT is but only 20.7% employed it, and at the same time 72.4% of the sample knew what HFUS is but only 34.5% employed it.

In the case of EIS and multispectral imaging, however, this double gap is more uniform, lacking both the knowledge and application components.

The problem of disconnection of clinical practice from diagnostic possibilities thus seems to have a dual motivation: cognizant and material.

### 4.2. Limitations of the Study

The purpose of our study was to verify the level of knowledge of these technologies and their effective use. The data provided by the study are interesting and highlight a phenomenon of deviation of clinical practice in plastic surgery from the state of the art in the diagnosis of skin neoformations.

However, our study has limitations related to the sample; in fact, on the one hand, the selection of plastic surgeons belonging to national and international scientific societies distributed over a wide geographic area and the choice of different health care and administrative contexts allows us to have a broad view of the problem; on the other hand, in order to have a global view, we would need to enlarge our sample.

In this context, these initial data of ours are intended to highlight a phenomenon and inspire further studies and actions aimed at bridging this gap between practice and theory.

## 5. Conclusions

In conclusion, the trends that emerged from our data suggest the need for better integration and communication between different disciplines. In particular, the creation of clinical pathways, dedicated treatment units, and common training paths [58,59] for multiple specialties in dermatosurgery could lead to a greater mixing of expertise and a more uniform and comprehensive patient care capability.

In the case of skin cancers, in fact, daily practice seems to be far from the possibilities that research offers us, although there is a good willingness on the part of clinicians for greater collaboration. The perspective for the future should be to combine diagnostic and surgical expertise and provide skin cancer patients with comprehensive, disease-centered care.

Hence, again, the importance of establishing the concept of comprehensive patient care, perhaps through the establishment of Skin Cancer Units.

## Figures and Tables

**Table 1:** Main application modalities and diagnostic indications.

Technique	Summary Indication
**Adhesive Patch Biopsy (APB)**	Early melanoma diagnostic determination (in association with clinic and dermoscopy) and prognostic information.
**Electrical Impedance Spectroscopy (EIS)**	Improves diagnostic ability of MSC and NMSC (in association with clinic and dermoscopy).
**Multispectral Imaging**	Complementary tool in the diagnostic definition of melanocytic lesions.
**High-Frequency Ultrasonography (HFUS)**	Useful in the diagnosis of surgical margins and determination of lesion depth.
**Optical Coherence Tomography (OCT)**	Very sensitive and specific instrument in the diagnostic determination of BCC. Useful in determining surgical margins and monitoring nonsurgical treatments.
**Reflectance Confocal Microscopy (RCM)**	Allows in vivo visualization of tissue at very high resolution. Allows determination of lesion margins and monitoring of nonsurgical treatments over time.
**Computer-Assisted Diagnosis and Artificial Intelligence (AI)**	Advanced methods with possibility of automation and standardization of the diagnostic process. Possible application on smartphones.

**Table 2:** Inclusion criteria.

Inclusion Criteria
Presence of a dermatology unit and a plastic surgery unit
Treatment of all malignant skin cancers except sarcomas
Complete responses to the questionnaire

**Table 3:** The extent of understanding and the accessibility of key diagnostic methods in oncodermatology among plastic surgeons.

	I Know It (%)	It Is Employed (%)
Adhesive Patch Biopsy (APB)	20.7	6.9
Electrical Impedance Spectroscopy (EIS)	6.9	0
Multispectral Imaging	6.9	0
High-Frequency Ultrasonography (HFUS)	72.4	34.5
Optical Coherence Tomography (OCT)	55.2	20.7
Reflectance Confocal Microscopy (RCM)	55.2	20.7
Computer-Assisted Diagnosis and Artificial Intelligence (AI)	72.4	13.8

**Table 4:** Analysis of the characteristics and limitations of diagnostic techniques.

Technique	Advantages	Limits	Sensibility	Specificity
Adhesive Patch Biopsy (APB)	Good sensitivity and specificity in the diagnosis of MSC. Can avoid unnecessary biopsies. Provides information on the genetic pattern of the lesion.	Do not use on bleeding or ulcerated lesions. Do not use on mucous membranes or palms of hands and feet. Some melanomas do not express the genes tested.	68.8–100% (Thomse [9]) - 91% (Gerami [10])	69.1–100% (Thomsen [9]) - 67% (Gerami [10]))
Electrical Impedance Spectroscopy (EIS)	Good sensitivity. Useful for monitoring lesions over time and diagnosing early melanomas, especially in patients with numerous pigmented neoformations.	Low specificity. Not usable in bleeding or ulcerated lesions. Many false positives in the presence of seborrheic keratoses. Efficacy and values vary depending on the thickness of the skin in the region analyzed.	MSC 96.6% (Malvehy [5])	MSC 34.4% (Malvehy [5])
Multispectral Imaging (MI)	Good sensitivity. Useful as a diagnostic aid in non-specialized settings.	Low specificity. Not sufficient to make diagnosis. Does not seem suitable for diagnosis of NMSC.	98.3% (Monheit [11])	9.9% (Monheit [11])
High-Frequency Ultrasonography (HFUS)	Good sensitivity. Useful in determining lesion depth. Useful in in vivo determination of lesions and in determining surgical margins.	Intermediate specificity. It is an operator-dependent method, difficult to estimate in cases of very thin or very thick tumors.	MSC 83–100% (Bessoud [12])	MSC 32% (Bessoud [12])
Optical Coherence Tomography (OCT)	Good sensitivity and specificity. Useful in defining surgical margins and monitoring noninvasive therapies. Useful in the diagnosis of BCC.	Lower resolution than RCM, does not reach a level of cellular resolution. Possible misdiagnosis in case of amelanocytic melanoma.	BCC 92.9% (Markowitz [13]) - MSC 74.1% (Gambichler [14])	BCC 80.0% (Markowitz [13]) - MSC 92.4% (Gambichler [14])
Reflectance Confocal Microscopy (RCM)	Good sensitivity and specificity. Provides very high-resolution images in vivo. Useful for determining margins and distinguishing different lesions based on specific patterns.	Depth of fabric examined limited. Elevated cost. Reduced efficacy in ulceration, inflammation, hyperpigmentation, and hyperkeratosis.	MSC 91–100% (Que [15]) - BCC 85–97% (Que [15]) - All skin cancer 95.3% (Borsari [16])	68–98% (Que [15]) - 89–99% (Que [15]) - All skin cancer 83.5% (Borsari [16])
Computer-Assisted Diagnosis and Artificial Intelligence (AI)	Broad potential, with possibilities for automating diagnostic processes, a field still largely unknown.	Field still unknown, difficulty in data standardization and deviation of results generated from real-world scenarios.	n/a	n/a

## Data Availability

The data presented in this study are available on request from the corresponding author. The data are not publicly available due toprivacy of research participants.
